# Philo-Medicus, on the Nature and Cure of Apoplexy

**Published:** 1802-05

**Authors:** 


### *To the Editors of the Medical and Phyfical Journal.*

GENTLEMEN,

THE following Remarks on the nature and cure of Apoplexy are offered to the public, not with a view to take any part in the prefent Controverfy on that fubject, but merely to afcertain how far certain modes of practice can be fupported by reafon and experience. If the hints thrown out could be of any fervice to the improvement of the healing art, I fhould feel myfelf highly gratified.

That Apoplexy is a preternatural determination of blood to the head is believed by all parties; and the diffection of bodies who have died of the difeafe confirms the opinion. But what is the caufe of this unequal diftribution of the blood? Are the veffels of the brain more apt to receive the blood when predifpofed to Apoplexy, than in health? Or, is there a partial obftruction of the circulation in any other part of the body? Of the firft of thefe we have no proof, of the latter we have many.

"After a *full meal*," fays Dr. Moffman, "Apoplexy very frequently occurs.† What effect does a full meal produce upon

† Medical and Phyfical Journal, Vol. vii. p. 208.

upon the ſanguineous ſyſtem? The Doctor anſwers, that "it increaſes the arterial impetus." If it increaſes the arterial impetus, does it do it in the whole ſyſtem or only in a part of it?* If it does it in the whole ſyſtem I cannot ſee how Apoplexy can follow as a conſequence. The circulation may be ſwifter, but ſtill it is equal through every part of the body. If it do it only in a part of the ſyſtem, Apoplexy may be induced; that is, if the circulation towards the head be increaſed, while that towards the extremities is diminiſhed, Apoplexy will moſt probably follow.

In what ſtate are the lower extremities of a perſon prediſpoſed to Apoplexy after a full meal? Are they not pale, cold, and feeble? Is not this occaſioned by ſome obſtruction of the deſcending aorta or other veſſels which carry blood to theſe parts? And may not this be occaſioned by an over-diſtenſion of the ſtomach and inteſtines? Thus a full ſtomach, by its ſtimulus, increaſes the arterial impetus, while at the ſame time, by its bulk, it obſtructs the flow of blood to the lower extremities. It may too by its preſſure upon the lungs prevent the return of the blood from the head. The conſequence of all theſe muſt be a preternatural determination to, and congeſtion in, the veſſels of the brain, hence Apoplexy.

Is it certain, or probable only, that in moſt caſes of Apoplexy there is effuſion from, or rupture of, the veſſels of the brain before death? How ſoon after death has any perſon opened the head of a patient who has died of Apoplexy? Is the force of the circulation ſo great as to rupture the veſſels of the brain when in a ſound ſtate? Or does a rupture only happen after the veſſels are become diſeaſed, or in a gangrenous ſtate from the continuance of over diſtenſion? Might not the blood and ſerum which have been found in many caſes have been thrown out after death, by the rupture of veſſels which had become gangrenous? Or may not the mouths of the exhalent veſſels be left in ſuch a manner, after death, as to pour out a quantity of ſerum? Is it not a common occurrence, in people who have died of other diſeaſes, to find quantities of blood or ſerum effuſed into the cellular membrane in different parts of the body ſoon after death? May not the effuſion in the brain have happened in the ſame way? So what we look upon as the cauſe of death may be the conſequence of it? I could wiſh much to ſee theſe points aſcertained; for if the force of the circulation be not ſufficient to rupture the veſſels of the brain when in a ſound ſtate,

---

* In this paper I have adopted the Socratical mode of reaſoning as being more laconic and pointed.

ſtate, and if no effuſion take place except in conſequence of diſorganization of the veſſels or gangrene, a material ſtep would be gained towards ſuccefsful practice. Why ſhould we doubt of the truth of theſe propoſitions till we ſee reaſons to the contrary?

If over diſtenſion of the ſtomach and inteſtines be the cauſe of obſtruction in the deſcending aorta and of compreſſion upon the lungs, and if obſtruction in the deſcending aorta and compreſſion on the lungs be the cauſe of a preternatural determination to the head, what is the indication of cure? To empty the ſtomach is the moſt natural. But how is this to be done? Emetics we are told are inadmiſſable, and evacuations by the anus are too ſlow in their operation, hence this cauſe of Apoplexy cannot in many inſtances be removed.

Why may emetics not be adminiſtered? Were they ever exhibited in a caſe of Apoplexy and did harm? Or is this merely an imaginary danger which no man ever ſaw? Is it merely the *ipſe dixit* of great men, handed from one to another, and now univerſally believed upon their authority without any other evidence? "Vomiting" ſays Dr. Cullen, "has been commended by ſome practitioners and writers; but *apprehending* that this might impel the blood with too much violence into the veſſels of the head, *I have never employed it.*" But is it certain that the operation of an emetic increaſes the flow of blood to the *brain*? If the anſwer be in the affirmative, I aſk, how is it aſcertained? The face indicates a preternatural quantity of blood in the head, but whether is this owing to the exertions of the perſon himſelf, ſtretching and twiſting the muſcles of the face in conſequence of the pain in the ſtomach; or is it a peculiar action of the emetic upon the ſtomach, producing a preternatural flow of blood to the brain? Was ever Apoplexy in perſons of the apoplectic habit brought on by taking an emetic? Is the face of a man vomiting after exceſſive intoxication, and when he has no marks of ſenſibility more than a perſon in Apoplexy, ſuffuſed in the ſame manner as one in ordinary circumſtances? So far as my obſervation goes, it is not the caſe; on the contrary I have ſeen them as pale as death. How, in caſes of this kind, could we determine whether or not there is a preternatural flow of blood to the *brain*? Did ever any of your correſpondents who condemn the uſe of emetics, ſee a perſon vomiting when in a fit of Apoplexy, and was his face additionally ſuffuſed with blood as in one taking a vomit for ſome other cauſe? Or do they condemn them merely from imaginary grounds, or by authority?

Is it not common to find relief from the exhibition of an emetic in caſes of head-ach? Does not the violent pulſation we feel in the head abate in many inſtances after an emetic, and and ſometimes even during the operation of it? How could this be the caſe if it increaſed the flow of blood to the head? Have we not all ſeen caſes of vomiting even where an emetic had been taken where the face was pale?

When any part of the body is violently exerted, is there not a preternatural flow of blood to that part? What reaſon have we to ſuppoſe an increaſed action of the brain during the operation of an emetic? And if there be no increaſed action of the brain, how do we prove, or imagine, that there is a preternatural flow of blood to it? Is it not more natural to ſuppoſe that the brain is the moſt quieſcent part of the whole body during vomiting, and that no preternatural ſuffuſion of blood will appear even in the face unleſs the perſon be in a ſtate of acute ſenſibility? Is it not more probable to ſuppoſe, ſince the thorax and abdomen with all their contents are thrown into a violent ſtate of action during the operation of an emetic, that the return of the blood from the head would be accelerated, and inſtead of being ſent back again, would be diſtributed to the parts of the body in action? Why ſhould we diſbelieve this till we ſee reaſons to the contrary?

If an over diſtenſion of the ſtomach and inteſtines be frequently a cauſe of Apoplexy, and if an emetic do not increaſe the flow of blood to the head, is it not the moſt rational application? But granting that it did increaſe the flow of blood to the head, if it did not immediately rupture the veſſels, would it not ultimately relieve the patient? If it rupture the veſſels, muſt they not have been previouſly in a diſeaſed ſtate from over diſtenſion, and death would have been the conſequence at any rate?

Beſides vomiting, the moſt effectual means of curing Apoplexy are bleeding and bliſtering. Bleeding, if the patient be of a full habit, which is generally the caſe, ought by no means to be omitted, and the ſooner it is done the better. Bleeding will not remove the cauſe of the diſeaſe, ſince it does not conſiſt in the quantity of the blood, but in the unequal diſtribution of it. It may, however, remove the agent which is likely to do the miſchief, or, as an ingenious friend of mine obſerves, it is like taking the bullet out of the gun before it is fired."

Bliſtering too is likely to be of ſervice; but how can it be performed ſo quickly as the urgency of the caſe requires? Could not the lower extremities, or any other part neceſſary be bliſtered by cloths dipped in boiling water? This would produce an effect in five minutes, which could not be done in twenty times as long by any other means which have been commonly uſed. The objections to its novelty, or its being cruel and painful, ought not to weigh a moment, ſince the patient is not ſenſible of the pain when it is applied, and the conſequences are not to be put in competition with the patient's life. Besides the quantity of serum extracted in this way, the stimulus of the boiling water would greatly increase the determination of the blood to the lower extremities; and if the obstructing cause be previously removed by an emetic, the patient would stand a fair chance to recover.

We have said that there is generally an over distension of the intestines as well as of the stomach, hence the propriety of using acrid injections to produce a copious evacuation by the anus as soon as possible. But this is not all the advantage attending injections of this kind; besides producing an evacuation by stool, they stimulate the whole course of the intestinal canal, and thus determine the blood to these parts. These observations will appear with more force, when we reflect that the suppression of an hæmorrhoidal flux has sometimes proved the cause of Apoplexy.

These remarks are not directed to the young practitioner as rules of practice, but rather to the *experienced*, to be *confirmed* or *refuted*. It is hoped that no arguments will be advanced that rest merely on authority. *I believe in no authority unsupported by reason and experience.* I am, &c.

*Scotland, March, 12, 1802* PHILO-MEDICUS.

P. S. I purpose, in my next, to give you a Review of the Theories and Doctrines of Cullen on the Nature and Cure of Apoplexy.

