# Errata

**Published:** 1802-07

**Authors:** 


					52o and 521, J or calx read calc.
521, for adeps reacl adep.
52 x, for creta read cretas.

				

